# Correction: Uncovering protein structure

**DOI:** 10.1042/EBC-2019-0042C_COR

**Published:** 2021-07-26

**Authors:** Elliott J. Stollar, David P. Smith

During the proofreading process an error in the text was missed which the authors would like to clarify. The authors apologise for any confusion arising from the error. In the section on Circular Dichroism, the paragraph beginning on page 668 the units should be in degrees rather than millidegrees and should read:

It is also standard practice for research papers to convert ΔAor θ into a value called mean residue molar ellipticity, [θ]_MR_,which takes into account the dependence on concentration, pathlength and controls for the number of residues in the protein (as mentioned above for ΔεMR). The historical units of [θ]_MR_ are degrees.cm^2^.dmol^−1^ and are equivalent to degrees.M^−1^.m^−1^ (which explains the factor of 100 in the equation below that converts pathlength units from centimetres into metres).

$${\left[\theta \right]}_{MR}\;=\frac{100\times \theta \;in\;degrees}{Molar\;concentration\;\times \;number\;of\;residues\;\times \;pathlength\;in\;cm}$$

In addition the y-axis of Figure 13 has been ammended to reflect this change and a clarifying sentence added.

**Figure 13:**
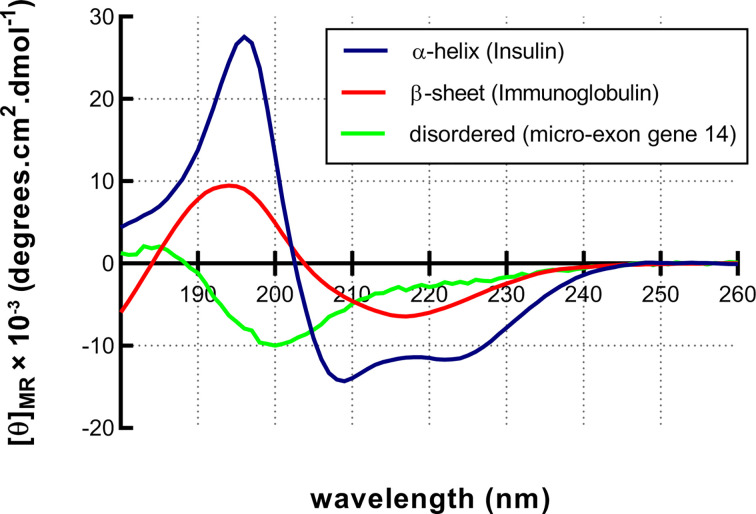
Characteristic CD spectra CD spectroscopy can be used to estimate the secondary structural content of a protein. Each secondary structural type has a characteristic spectrum. α-helical proteins like Insulin (blue) have a double hump spectrum with peaks at negative bands at 222 and 208 nm and a positive band at 193 nm. Proteins with well-defined antiparallel β-sheets like Immunoglobulins (red) have negative bands at 218 nm and positive bands at 195 nm. Disordered proteins such as the micro-exon gene 14 (green) have very low signal above 210 nm and negative bands near 195 nm. The [θ]_MR_ values are large and before plotting them, they are often divided by 1000 to make the y-axis scale more appropriate, this change is reflected in the units where [θ]_MR_ is multiplied by 10^−3^ to maintain the original values.

